# Cell membrane-based nanomaterials for theranostics of central nervous system diseases

**DOI:** 10.1186/s12951-023-02004-z

**Published:** 2023-08-19

**Authors:** Wenyue Li, Junwei Cheng, Fangfei He, Peisen Zhang, Ni Zhang, Jian Wang, Qiliang Song, Yi Hou, Zhihua Gan

**Affiliations:** 1 College of Materials Science and Engineering, College of Life Science and Technology, Beijing University of Chemical Technology, Beijing, 100029 China; 2 Department of Psychiatry, West China Hospital of Sichuan University, Chengdu, 610041 China; 3 Department of Head and Neck Surgery, National Cancer Center/National Clinical Research Center for Cancer/Cancer Hospital, Chinese Academy of Medical Sciences, Peking Union Medical College, Beijing, 100021 China; 4 Shandong Peninsula Engineering Research Center of Comprehensive Brine Utilization, Weifang University of Science and Technology, Shouguang, 262700 China

**Keywords:** Cell membrane, Nanomaterials, Central nervous system diseases, Theranostics

## Abstract

Central nervous system (CNS) diseases have been widely acknowledged as one of the major healthy concerns globally, which lead to serious impacts on human health. There will be about 135 million CNS diseases cases worldwide by mid-century, and CNS diseases will become the second leading cause of death after the cardiovascular disease by 2040. Most CNS diseases lack of effective diagnostic and therapeutic strategies with one of the reasons that the biological barrier extremely hampers the delivery of theranostic agents. In recent years, nanotechnology-based drug delivery is a quite promising way for CNS diseases due to excellent properties. Among them, cell membrane-based nanomaterials with natural bio-surface, high biocompatibility and biosafety, are of great significance in both the diagnosis and treatment of different CNS diseases. In this review, the state of art of the fabrication of cell membranes-based nanomaterials is introduced. The characteristics of different CNS diseases, and the application of cell membranes-based nanomaterials in the theranostics are summarized. In addition, the future prospects and limitations of cell membrane nanotechnology are anticipated. Through summarizing the state of art of the fabrication, giving examples of CNS diseases, and highlighting the applications in theranostics, the current review provides designing methods and ideas for subsequent cell membrane nanomaterials.

## Introduction

Central nervous system (CNS) diseases, craniocerebral and spinal cord diseases, have been widely acknowledged as one of the major health concerns globally. There will be 135 million cases worldwide by mid-century, predicted by the non-profit group Alzheimer’s Disease International [1]. The number of neurological disease cases has been gradually increasing with the progressive rise in life expectancy [2], because the neurotrophic signalling in sensory neurons will attenuate as in aging leading to the decreased nervous system function [3]. As many countries’ populations get older, CNS diseases will overtake cancer to become the second leading cause of death after cardiovascular disease by 2040, predicted by the World Health Organization (WHO) [1]. CNS diseases can be classified into two categories, the one is acute brain injury including stroke, cerebral ischemia (CI), brain injury (BI), and epilepsy, and the other is chronic neurodegenerative diseases (NDs) including Alzheimer’s disease (AD), Parkinson’s disease (PD), Huntington’s disease (HD) [4]. These diseases acutely impair the memory and cognition, speaking ability, moving ability, and even breathing ability [5].

Most CNS diseases lack of effective diagnostic and therapeutic strategies. For example, CNS tumors are removed by craniotomy, with high risks and severe irreversible damages to the brain. Additionally, chemotherapy and radiotherapy are the common methods to treat cerebral neoplasms but with serious side effects such as cell damages and skin irradiation [6]. Few curative methods are available for CNS diseases, without delaying or reversing disease progression. As a result, it is an urgent need to develop specific diagnostic and therapeutic strategies with early intervention for CNS diseases.

Some new drugs have been developed for CNS diseases but limited by the blood-brain barrier (BBB), which is a physical and metabolic barrier restricting transportation between the blood and neural tissues, leading to low efficiency for delivering drugs to the brain [6–8]. There exist the physical barrier and the biochemical barrier in the brain. Endothelial cells are bound together with tight junctions, surrounded with basement membrane composed of peripheral cells and the matrix, and the perivascular foot of many astrocytes encircles the outermost, co-forming a multi-layer membrane protective structure of brain capillaries, that is, the physical barrier, which can hinder the transportation of cargos through the paracellular passage between the adjacent endothelial cells [9]. In addition, the biochemical barrier with a lot of transporters and enzymes strictly controls materials transportation as well [7]. Therefore, BBB can shield the CNS from neurotoxic substances circulating in the blood but limit the effective transportation of most of substance such as drugs, nucleic acids, proteins, imaging agents and other macromolecules, which lead to few ad hoc therapeutic ways available to reach exact targets in the brain for prognosis [10, 11].

Accordingly, the drug design for the CNS diseases faces with a dilemma. On one hand, low-molecular-weight lipophilic drug molecules can passively traverse the BBB, however, the fast urinary clearance largely hamper the bioavailability; On the other hand, larger lipophilic drugs with molecular weight greater than 400-500 Da usually fail to cross the BBB in pharmacologically significant quantity [12, 13]. In this context, only a few drugs penetrating the BBB are currently approved by the Food and Drug Administration (FDA) to improve disease but cannot predict disease progression and complete treatment [14, 15].

For example, tacrine, donepezil, rivastigmine, galantamine, and memantine approved by the FDA for AD treatment could provide symptomatic relief, temporarily enhancing cognitive ability, but could not block the progression of the disease, with by-effects of nausea, vomiting, diarrhoea, etc. [15, 16].

To effectively overcome the BBB obstacles, some strategies such as changing permeability efficiency of barriers, using carrier and receptor-mediated drug delivery have been adopted [17]. Nanotechnology-based drug delivery is a quite promising way owing to excellent physical and chemical properties of nanomaterials such as appropriate size (from 0.1 to 100 nm), large specific surface area [18–20]. Additionally, the brain capillary endothelial cells tend to transport and internalize nanomaterials *via* endocytosis and transcytosis mechanism easily [21]. To date, several nanomaterials have been applied for CNS diseases in laboratory studies and preclinical trials, such as organic and inorganic nanomaterials, polymers nanoparticles, carbon-based nanomaterials, liposomes, and metal nanoparticles [4, 22]. Nevertheless, biocompatibility, undesirable pharmacokinetics and cytotoxicity of nanomaterials in curing CNS diseases are controversial [23].

In recent years, cell membrane-based nanomaterials have attracted extensive attention owing to natural bio-surface, high biocompatibility and biosafety, especially in the field of theranostics of CNS diseases. Comparing with other nanomaterials, the natural biointerfacing capabilities of cell membranes endow the nanomaterials with not only the ability to disguise themselves as intrinsic cells to escape from the immunological surveillance in vivo [24], which can extend blood circulation time, but also the various biological function of cell membrane such as low toxicity [25], targeting [26] and BBB penetrating [27].

In this review, the state of art of the fabrication of cell membranes-based nanomaterials is introduced. The characteristics of different CNS diseases are briefly introduced and the application of cell membranes-based nanomaterials in the theranostics of them are summarized. In addition, the future prospects and limitations of cell membrane nanotechnology are anticipated. Through summarizing the state of art of the fabrication, giving examples of CNS diseases, and highlighting the applications in theranostics, the current review provides designing methods and ideas for subsequent cell membrane nanomaterials.

## Cell membrane-based nanomaterials

To date, a variety of nanomaterials have been developed for the theranostic applications of CNS diseases. Nevertheless, nanomaterials still have to face many difficulties. Specifically, the conventional nanomaterials will readily be captured by immune systems, which are difficult to penetrate BBB and release drugs [26]. It is worth noting that certain cells such as monocytes, macrophages, neutrophils, etc. could be selectively delivered into the brain and not captured by immune systems [7]. In this context, cell membrane-based nanomaterials, which are fabricated through the combination of various nanomaterials and cell membrane, can largely overcome the demerits of the conventional nanomaterials.

At present, the main design concept of cell membrane-based nanomaterials is cell membrane coating. Through this strategy, the intrinsic properties of cell membrane can be replicated on the nanomaterials, such as the stealth nature to immune systems, specific targeting to inflammatory site, penetrating ability to biological barriers, and so on [28]. With these biological functions, the cell membrane-based nanomaterials can not only possess higher biocompatibility but also have better diagnosis and treatment efficacy in vivo [29].

### Design and synthesis of cell membrane-based nanomaterials

**Scheme 1:**
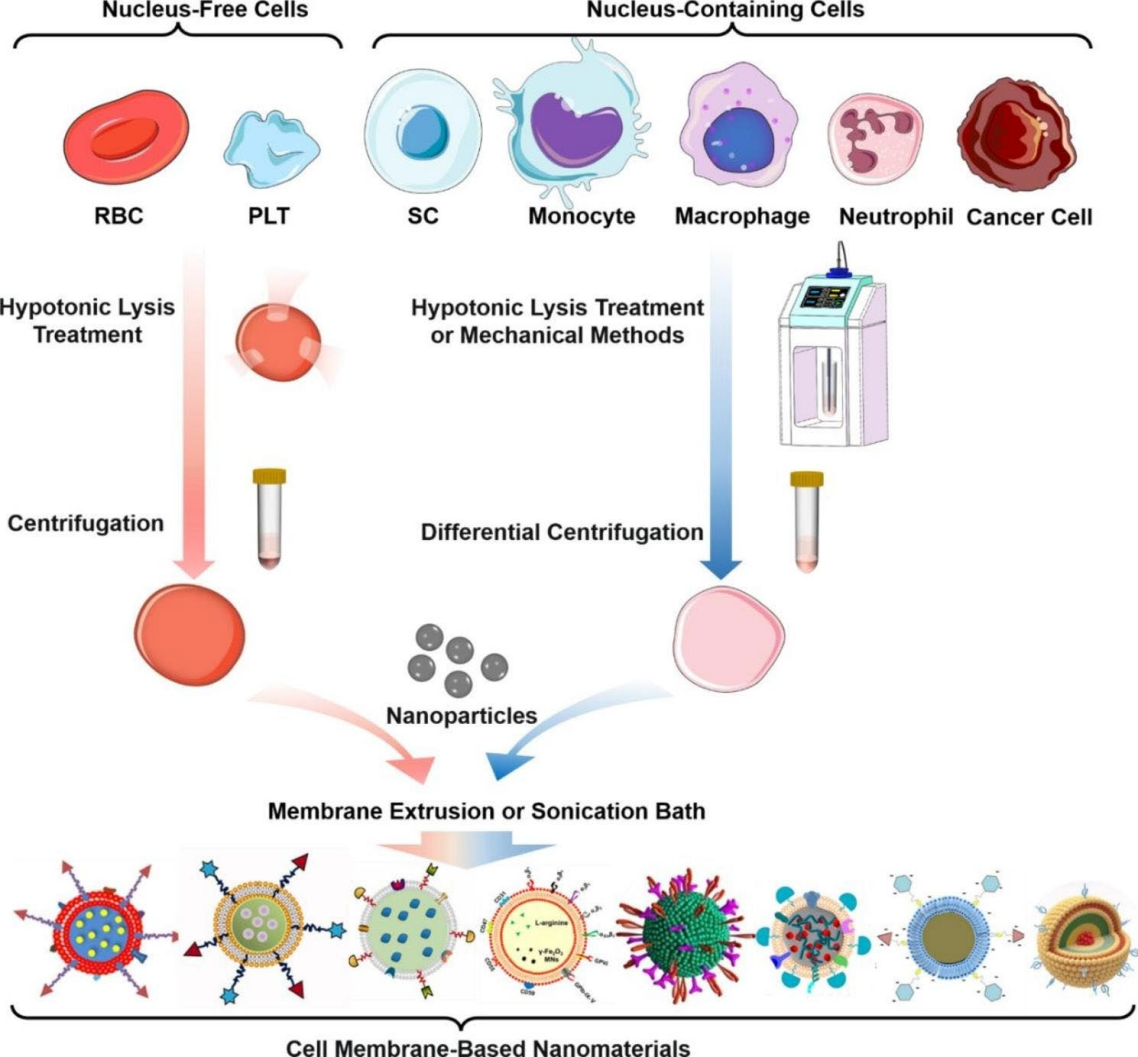
The schematic illustration of the synthesis procedures of cell membrane-based nanomaterials

According to the previous studies [25], the synthesis approaches of cell membrane-based nanomaterials are similar, which can be mainly divided into three procedures (Scheme 1), i.e., (1) cell membrane extraction, (2) inner nanoparticles preparation, (3) the fusion process of cell membrane and nanoparticles.

#### Cell membrane extraction

Generally, the cell membrane extraction procedure mainly includes two steps, that is, cell lysis and membrane purification. Specifically, hypotonic treatment is usually used to lysis cells. After that, the repeated freeze-thawing, multiple sucrose gradient centrifugation and differential centrifugation are usually adopted to removing the nucleic and cytoplasmic contents of the cells. Finally, the specific buffers or extrusion are further employed to purify the resultant cell membranes [28, 30].

The cell membrane of nucleus-free cells is the easiest to be extracted. For example, red blood cells (RBCs) are one of the earliest types of cells which are used to extract cell membranes for particles coating. They can be readily obtained from the whole blood through centrifuged approach. RBCs can be lysed by a hypotonic lysis treatment (sometimes accompanied by ultrasonic vibration) to release the intracellular materials. RBC membranes can thus be obtained through simple centrifugation [31].

The acquirement of the cell membrane from nucleus-containing cells are relatively difficult in comparison with nucleus-free cells, especially for those tiny sized cells with highly specialized surface proteins [22]. Nucleus-containing cells can be lysed by hypotonic treatments combining with repeated freeze-thaw cycles and mechanical methods including a homogenizer or ultrasonication [32, 33]. Next, differential centrifugation or discontinuous sucrose gradient centrifugation can be utilized to remove intracellular contents and obtain the membranes [32]. For example, ultrasonication and multiple differential centrifugations have been employed to lyse cells, and remove the nucleus and cytoplasm in the extraction of neutrophil membrane. The ultrasonication and centrifugal rotational speed must be controlled precisely.

#### Nanomaterials fabrication

At present, various types of nanomaterials, mainly including liposomes, inorganic nanoparticles, and polymer nanoparticles have been widely utilized as the drug assisting delivery tools. The conventional drug molecules, such as curcumin or doxorubicin (DOX), can be readily loaded in the nanoparticle through covalent or noncovalent interaction. All such nanoparticles can be served as the inner core of the cell membrane-based nanomaterials [28, 34, 35]. Apparently, choosing different nanomaterials, and paring with the more appropriate cell membrane, can target the disease site, maximize the diagnosis and treatment effect of nanomaterials for CNS diseases.

#### Fusion between cell membrane and nanomaterials

After cell membrane extraction and nanomaterials fabrication, fusion is essential to fabricate an effective cell membrane-coated nano-agents as the last procedure. The most common method reported of the fusion process is membrane extrusion or sonication bath to coat the core nanomaterials with cell membranes [24]. For the membrane extrusion process, porous polycarbonate membranes with different pore size are usually employed to produce the cell membrane-based nanomaterials through the mechanical forces of multiple squeeze cycles [36]. During the extrusion process, the mechanical force applied destroys the membrane structure, and the repeated extrusion process facilitates the fusion of cell membrane onto nanoparticles. Multiple passes processes could be employed to control the size and multilamellarity of extruded cell-membrane based nanomaterials whose sizes depend on the pore size. This way requires greatly large forces during extruding hard materials via a porous membrane [37]. Generally, the porous polycarbonate membranes with large aperture are prioritized to prepare nanomaterials and then a small aperture membrane is changed to reduce the size. It is reported that the size of the resultant materials could be changed slightly by the extrusion pressure as well. The larger the extrusion pressure, the slightly smaller the size [38]. Apart from the extrusion, ultrasound can also be adopted to encapsulate nanomaterials into cells membrane through disrupting the lipid bilayer. Subsequently, the cell membranes and the nanomaterials would attracted each other to form a spherical shape so as to achieve minimum entropy [39]. However, through the sonication process, the functional proteins of cell membrane may be denatured by the excessively high temperature, which severely impacts their biological performance. In addition, the obtained cell membrane-based nanomaterials exhibit poor size uniformity in comparison with the extrusion. Therefore, it has to be admitted that sonication bath is a feasible approach to obtain the cell membrane-based nanomaterials with higher yields. However, the sonication power should be controlled more precisely [28, 40]. Apart from the membrane extrusion and sonication, other novel technologies have also been reported to induce nanoparticles into cell membrane, such as the microfluidic-electroporation technique [37]. Microfluidic-electroporation technique is employed by external electrical field to form multiple transient pores in the cell membrane, promoting the entry of nanomaterials. This method can prepare uniform biomimetic cell membrane-based nanomaterials with high reproducibility [41]. Some researchers also attempted to directly ingest nanoparticles into cells through co-incubation, and this method has been used to deliver drugs into the brain [42–44], such as graphene nanoplatform-mediated cell membrane coating technique [45].

### Types of cell membranes

To date, versatile cells such as RBCs, platelets (PLTs), monocytes, neutrophils, macrophages and stem cells (SCs) have been investigated for preparation of cell membrane-based nanomaterials for CNS diseases [29, 46].

#### RBC membrane

RBCs are the most abundant cells that are responsible for the oxygen and carbon dioxide transport and the most common cells used to fabricate cell membrane-based nanomaterials [25, 28]. The protein CD47 on the RBC membrane prevents nanomaterials from the mononuclear phagocyte system (MPS) resulting in RBCs having the nonimmunogenic property with more circulation time in vivo [47].

#### PLT membrane

PLTs, a type of small enucleated blood cells that can migrate to the injury site inhibiting excessive bleeding, are implicated in haemostasis and arterial thrombosis [48]. This characteristic endows PLTs with unique targeting adhesion to the damaged blood vessel. Beneficial from this merit, in the previous studies, the PLT membrane has been adopted to construct nanomaterials with active targeting abilities to various hemorrhage related diseases, such as thrombus, vascular inflammation, brain hemorrhage, etc. PLT membrane-coated nanoparticles are considered as one of the potential targeting probes for ischemic stroke, which can be used for thrombus targeting [49, 50].

#### Monocyte membrane

Monocytes are the largest white blood cells in vivo, which possess strong phagocytic ability and can be derived from hematopoietic stem cells in the bone marrow. Owing to the characteristics that monocytes can defense against intracellular pathogenic bacteria and parasites, and recognize and kill abnormal cells, this type of cells have been frequently used as the design of drug delivery system with active targeting ability [51]. Monocytes can not only effectively prolong the circulation period of conventional drugs in blood stream due to reduced opsonization and self-recognition mechanisms, but also help the drugs penetrate the vascular endothelium into lesion tissues [24]. Therefore, monocytes can be regarded as one of the satisfactory choices to construct the cell membrane-based nanomaterials.

#### Macrophage membrane

Macrophages are a kind of innate immune cells derived from monocytes, which phagocytize cellular debris and pathogens, and activate lymphocytes or other immune cells to respond to pathogens [52]. Taking charge of the detection, phagocytosis and breakage of “invaders” in vivo, macrophage membrane-encapsulated nanoparticles have played an increasingly popular role in targeting inflammatory diseases [28]. Owing to the over-expression of integrin lymphocyte function-associated antigen 1 (LFA-1) of macrophages, macrophage membrane-encapsulated nanoparticles can bind to intercellular cell adhesion molecule 1 (ICAM-1) on brain endothelial cells and easily crossing the BBB [53]. In addition, macrophage membrane-encapsulated nanoparticles can accumulate in the injury site due to the interaction between integrin α4 and Mac-1 on the macrophage membranes and vascular cell adhesion molecule-1 (VCAM-1) on impaired microvascular endothelium, achieving a higher drug delivery efficiency [54]. Moreover, macrophages can adapt to the constantly alternative environment by changing their morphology and physiological function, called macrophage polarization [55]. The cell membrane from different phenotype of polarized macrophage possesses distinct bio-function, which largely expand the bio-application of the macrophage membrane.

#### Neutrophil membrane

Neutrophils are the most abundant type of granulocyte, making up 40–70% of all white blood cells. Neutrophils can sense and transfer to the inflammatory site, and penetrate BBB to the inflamed brain region [56]. This active inflammation targeting ability has been used for the nanomaterials design [28]. Moreover, nanoparticles delivery relying on neutrophils has been widely utilized for blocking immune recognition. However, some studies have demonstrated that neutrophils can be activated and subsequently release reactive oxygen species, bioactive lipid mediators and neutrophils extracellular traps (NETs) at the inflamed lesion site, leading to inflammatory damage [57], which should be considered when designing the neutrophils based nanomaterials.

#### SC membrane

SCs are cells with infinite or immortal self-renewal capacity, capable of multidirectional differentiation. SCs such as mesenchymal stem cells (MSCs) and neural stem cells (NSCs) are confirmed to possess intrinsic tumor-homing capacity, and inherent capacity to track malignant cells over long distances in the brain. On this basis, SCs have been utilized to constructed nano-based drug delivery systems to carry conventional drugs [58, 59]. Moreover, in vitro study demonstrated that MSCs can easily be isolated, propagated, and autologously transplanted into patients, thus the immune exclusion reaction may be largely reduced *via* this strategy with autologous reinfusion [60, 61].

#### Cancer cell membrane

Cancer cell membrane is also a promising ingredient for nanoparticles design due to its excellent characteristic such as robustness, source cell-specific targeting, antigenic display and immune escape, etc. As is known, with the indefinite proliferation of tumor cells, the membrane can be readily extracted. With the stealth nature, the tumor cells membrane can endow nanomaterials with immune escape capacity. In addition, with homing effect, the tumor cells can actively target the tumor lesions. Therefore, cancer cell membranes can provide a good idea for the nanoparticle design on the field of the tumor diagnosis and treatment [24, 62, 63].

## CNS Diseases

CNS diseases are difficult to cure, comprising varied types such as Alzheimer’s disease (AD), Stroke, Spinal Cord Injury (SCI), Parkinson’s disease (PD) and CNS tumors. Owing to the aforementioned excellent properties, cell membrane-based nanomaterials have been used as an ideal tool for the theranostics of CNS diseases.

### AD

AD is one of the most common CNS diseases, with mental, behavioral and functional impairment, and loss of learning as cognitive performance deteriorates [64, 65]. According to WHO, the number of people living with dementia estimated to stand at 55 million in 2019 and will rise to 139 million in 2050 [66]. At present, it is widely accepted that the hyperphosphorylation of tau protein is closely related to the development of AD. The abnormal hyperphosphorylation has a high tendency to aggregate, which falls off from the neurocytoskeleton inducing intracellular neurofibrillary tangles [67–69]. Beyond that, reactive oxygen species (ROS)-induced neuronal mitochondrial dysfunction and excessively activated microglia-liberated numerous inflammatory factors as well as neurotoxic substances are the significant causes [69, 70].

Unfortunately, even if different therapeutic strategies have been developed, AD is still an incurable, chronic and disabling disease. At the advanced stage of AD, the clinical outcomes of the current clinical drugs, such as acetylcholinesterase inhibitors (rivastigmine, galantamine, donepezil) and N-methyl d-aspartate receptor antagonist, are limited [65]. According to the recent studies, the poor delivery of these drugs is one of the main reasons to cause the unsatisfactory efficacy [71]. Under this circumstance, many studies have been developed to explore new drugs delivery strategies.

**Fig. 1:**
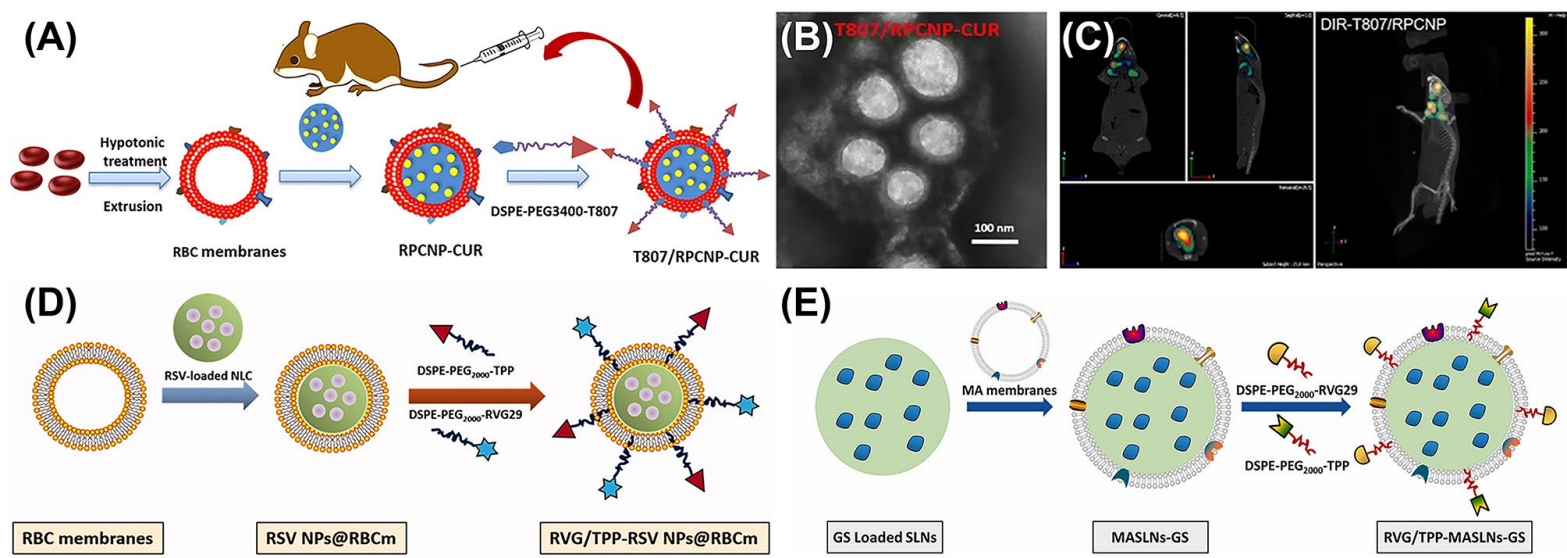
(**A**) The preparation of T807/RPCNP-CUR by using RBC membrane. (**B**) TEM image of T807/RPCNP-CUR. (**C**) in vivo brain-targeting ability of T807/RPCNP-CUR in AD model mice determined by an IVIS® Spectrum. (**D**) The preparation of RVG/TPP-RSV NPs@RBCm. (**E**) The preparation of RVG/TPP-MASLNs-GS. Reproduced with permission from Gao et al. [35], Han et al. [70], Han et al. [73]

Gao et al. [35] fabricated T807/RPCNP-CUR (Fig. 1A) that could cross the BBB and localize p-tau in neurons for AD therapy. The RBC membrane is employed to coat PLGA particles (RPCNP) that are loaded with curcumin (CUR). T807 molecules, a novel tau positron emission tomography imaging agent for AD, that can cross the BBB and bind to phosphorylation tau positive human brain sections are embedded on the surface of the RBC membranes. According to the transmission electron microscope images, RBC membranes are successfully coated on the surface of T807/RPCNP-CUR nanomaterials as shown in Fig. 1B. To confirm the in vivo brain-targeting ability, the okadaic acid (OA)-induced AD mouse model was adopted as the animal model. After intravenous injection of the near-infrared dye DIR-tagged T807/ RPCNP-CUR, strong fluorescence signal can be observed in the brain in vivo (Fig. 1C), suggesting that T807/RPCNP-CUR successfully penetrate the BBB and accumulate in the brain area. Overall, T807/RPCNP has a high affinity with hyperphosphorylated tau in nerve cells, which can reduce p-tau levels and suppress neuronal-like cells death in vitro and in vivo to relieve AD symptoms such as the memory impairment improving. In another study, to augment the anti-AD therapy efficacy, this research group [72] selected triphenylphosphine (TPP) molecules, a kind of molecules that can enter mitochondria by exploiting the negative mitochondrion membrane potential, as the mitochondrion targeting ligand. With the help of TPP molecules, the T807/RPCNP system possesses the ability to target neuronal mitochondria with higher specificity binding to neurons for AD treatment.

Han et al. [70] devised a dual-modified biomimetic nano-system (RVG/TPP-RSV NPs@RBCm) (Fig. 1D). The RBC membrane is used to coat the nanostructured lipid carriers (NLC), which are loaded with resveratrol (RSV) as a model antioxidant. The rabies virus glycoprotein (RVG29) and TPP molecules are attached to the RBC membrane surface. RVG/TPP-RSV NPs@RBCm can accumulate in the neuron after penetrating the BBB with the navigation effects of RVG, target the mitochondria efficiently with the assistant of TPP, and finally deliver the drugs to the key pathologic site effectively protecting neurons. In another publication, Han et al. [73] employed the macrophage membrane as the shell to coat both the solid lipid nanoparticles (SLNs) and Genistein (GS) for efficiently delivering GS into neuronal mitochondria. The RVG29 and TPP are attached to the surface of macrophage membrane (RVG/TPP-MASLNs-GS) (Fig. 1E). Beneficial from the great biocompatibility and immunological characteristics of macrophages membrane, SLNs can avoid to be quickly cleared by reticuloendothelial system (RES) elimination to a great extent. Additionally, RVG/TPP-MASLNs-GS can cross the BBB and selectively target to the neurons due to TPP and RVG, and accurately release GS to relieve AD symptoms in vivo.

### Stroke

Stroke is one of the leading causes of death worldwide, which is mainly including ischemic stroke and hemorrhagic stroke. It is reported that the global number of Ischemic stroke deaths increased from 2.04 million to 3.29 million between 1990 and 2019, and will increase to 4.90 million by 2030 [74]. This kind of CNS disease usually manifests as a sudden or rapid onset of loss of function of a specific part of the body due to the brain, retina or spinal cord damage [75]. The causing of stroke is relatively complicated. Specifically, ischemic stroke may be induced by stenosis and occlusion of intracranial and intracranial arteries, cerebral artery embolism, hemodynamic factors, hematological factors, inflammation, infection, etc.; while hemorrhagic stroke may usually be provoked by the aneurysm rupture, cerebral artery malformation, trauma and other factors [2, 76]. Approximately one-third of stroke victims die within a year, and a similar number of patients are permanently incapacitated [75].

**Fig. 2:**
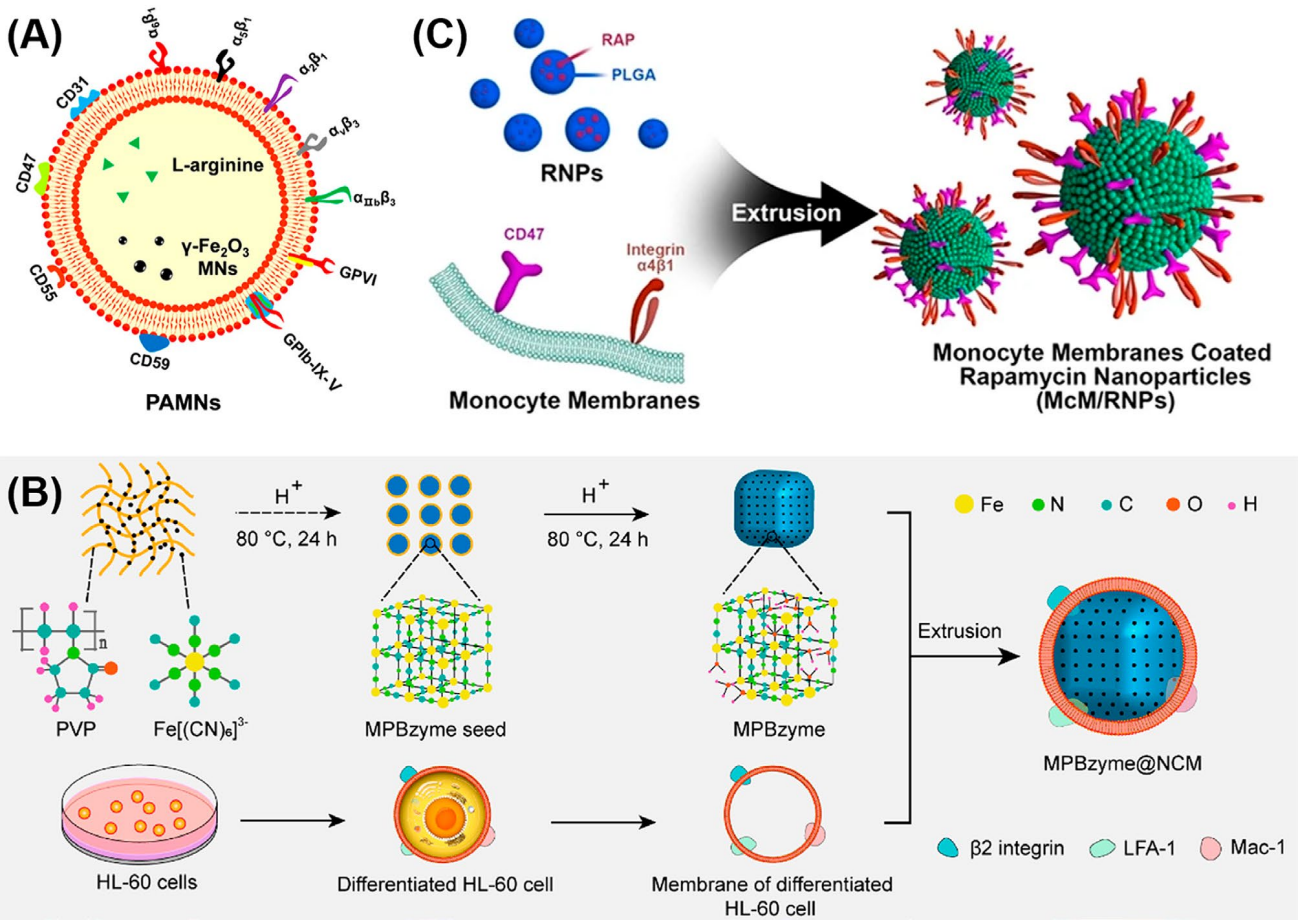
(**A**) Schematic diagram of PAMNs structure. (**B**) Illustration of MPBzyme@NCM preparing process. (**C**) Schematic drawing of McM/RNPs fabrication. Reproduced with permission from Li et al. [49], Feng et al. [27], Wang et al. [77]

Li et al. [49] prepared a biomimetic nanomaterial by loading the l-arginine and γ-Fe_2_O_3_ magnetic nanoparticles (PAMNs) into PLT membrane, as shown in Fig. 2A. Under the guidance of an external magnetic field and the natural adhesion capability of PLTs to plaque, PAMNs can target the ischemic area in a mouse model of cerebral, cortical ischemic stroke rapidly, and release l-arginine at the thrombus site to promote vasodilation. In addition, both endothelial cells and fresh natural PLTs produce nitric oxide (NO) at the thrombus site leading to the recovery of blood flow, disruption of the local PLTs aggregation, and reperfusion of the stroke microvascular as well. As a result, PAMNs successfully remodel the microvasculature network around the injury and recover blood flow to the lesion indeed relying on the natural thrombus-targeted adhesion capability of the PLT membrane and the magnetic field responsiveness of γ-Fe_2_O_3_ magnetic nanoparticles (MNs), which are clearly confirmed by the high-resolution, multimodal optical imaging and cerebral blood flow measurement. Feng et al. [27] designed a noninvasive targeted delivery system (MPBzyme@NCM) (Fig. 2B) for ischemic stroke treatment. The mesoporous Prussian blue nanozyme (MPBzyme), which possesses robust anti-inflammatory and antioxidative stress properties, is encapsulated by neutrophil-like cell membrane (NCM). MPBzyme@NCM can successfully target the damaged brain by the interaction between neutrophil-like cell membrane and the inflamed brain microvascular endothelial cells. After the ingestion of MPBzyme@NCM, the microglia would polarize toward M2 phenotype, thereby reducing the recruitment of neutrophils and decreasing apoptosis of neurons, resulting in a long-term therapeutic efficacy against ischemic stroke. Wang et al. [77] reported a functionalized nanoparticles (McM/RNPs) that are prepared by coating rapamycin nanoparticles (RNPs) with monocyte membranes (McM) (Fig. 2C) for avoiding reperfusion-induced injury in ischemic stroke. The obtained McM/RNPs can effectively target and bind to inflammatory endothelial cells and simultaneously inhibit the adhesion between monocytes and endothelium, decreasing uncontrollable inflammation. In addition, the functionalized nanoparticles can reach the lesion site and release rapamycin (RAP) drug inhibiting microglia proliferation, relieving inflammation and improving the neural regeneration of transient middle cerebral artery occlusion (tMCAO) rats.

### SCI

As a neurological disorder, SCI is usually caused by the traumatic injury, referring to the complete or partial loss of spinal nerve function. It may lead to serious consequences, from some limb sensory abnormalities and urinary incontinence to paraplegia, and even brain death. It is reported that the incidence of the traumatic spinal cord injury is 10.5 cases per 100,000 people, and the global annual estimated 768,473 new cases [78]. SCI are irreversible accompanied by several complications such as neurogenic bladder and bowel, urinary tract infections, pressure ulcers, orthostatic hypotension, fractures, deep vein thrombosis, spasticity, autonomic dysreflexia, pulmonary and cardiovascular problems, and depressive disorders [79, 80]. Just like the BBB, the blood-spinal cord barrier (BSCB) is a thorny barrier for drug delivery of SCI. The better drug deliver strategy that can cross the BSCB and effectively treat the SCI is much needed [53].

**Fig. 3:**
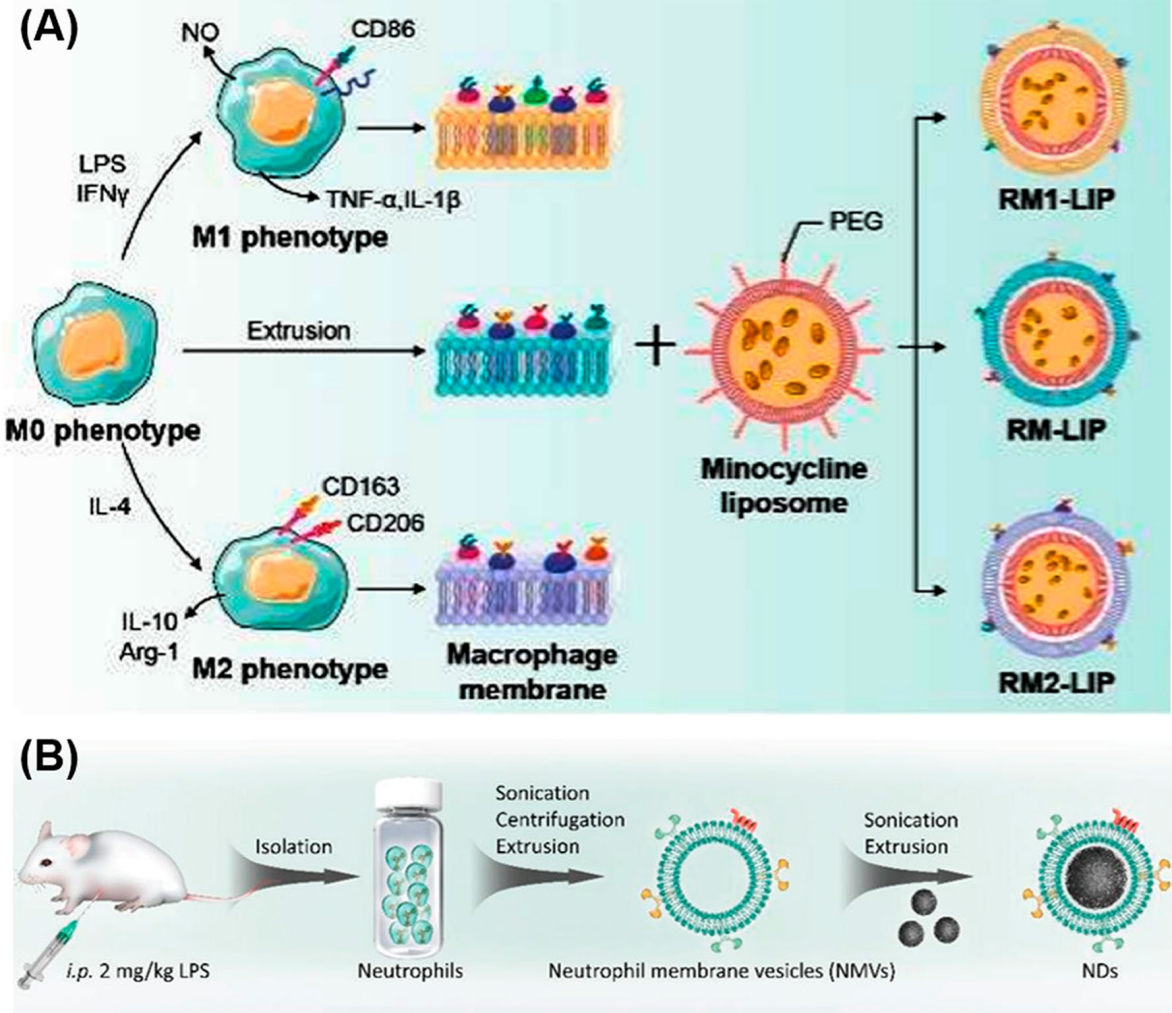
(**A**) Preparation of different polarized macrophage membrane-camouflaged liposomes (RM-LIPs). (**B**) Fabrication process of the neutrophil decoy (ND). Reproduced with permission from Tang et al. [54], Bi et al. [57]

Yu et al. [53] prepared an efficient therapeutic system (Met-CNG-GSH) by loading the metformin (Met) into the glutathione-modified macrophage-derived cell membrane-encapsulated nanogels (CNG-GSH). Such nano-system can cross the BSCB and treat the SCI effectively. Through in vivo imaging validation, Met-CNG-GSH accumulates at the lesion site with a great targeting capacity. In addition, the animal experiments demonstrated that Met-CNG-GSH had a good therapeutic effect in alleviating oxidative stress, suppression of inflammation and apoptosis, which significantly improve the motor function recovery of SCI mice models. Tang et al. [54] reported a type of macrophage membrane-camouflaged liposome (RM-LIPs) which can better escape from the host immune systems and actively accumulate at the trauma site of SCI mice models. Particularly, through loaded with minocycline, RM-LIP system (RM-LIP/MC) (Fig. 3A) can remarkably inhibit the inflammation of injury sites and exhibit an outstanding therapeutic effect on SCI mice. In another work, Bi et al. [57] proposed a nano-decoys strategy to promote functional recovery of SCI *via* reducing the infiltration of immune cells (such as neutrophils). In typical, the neutrophil decoy (ND), which prepared by coating the polydopamine (PDA) nanoparticles with neutrophil membrane (Fig. 3B), possessing strong anti-oxidative and anti-inflammatory capacities to clean excessive reactive oxygen and nitrogen species. During the ND treatment, the infiltration of neutrophils was demonstrated to be effectively reduced, leading to the relief of the inflammatory and oxidative state of the microenvironment in contusion mice model of SCI. Through this strategy, neural function is expected to be restored.

### PD

PD is another customary CNS disease, which is induced by the loss of dopaminergic neurons in the pars compacta of the substantia nigra. Generally, the symptoms of PD mainly including tremors, bradykinesia, muscle rigidity, difficulty with postural balance, accompanied by mental disorder, dementia, and memory impairment [81]. More frighteningly, it is relevant with a high disability rate [82]. According to the statistics, there are 3 million elderly patients affected by PD in China in 2020, and the number of patients with all ages will be up to 5 million by 2030 [83, 84]. In clinic, therapeutic strategies including chemotherapy and surgical treatment can relieve the symptoms of patients. Nevertheless, the former has unavoidable side effects, while the latter is an invasive therapy. In addition, currently available treatments cannot predict and modify the evolution of the disease. In this context, the cell membrane-based nanomaterials can serve as a better candidate for PD theranostics, which has the potentials to realize the noninvasively and lower-side-effect treatments [85, 86].

Liu et al. [86] coated Cu_2-x_Se- poly(vinylpyrrolidone) (PVP)-Quercetin (Qe) nanoparticles with the membrane of MES23.5 neuronal cells to produce the nano-drugs, known as CSPQ@CM nanoparticles. Through a series in vitro and in vivo experiments, CSPQ@CM nanoparticles can target toward microglia specifically through the interactions between the surface α4β1 integrin of microglia and vascular cells adhering to molecule-1 (VCAM-1) expressed on the surface of neuronal cells. In addition, CSPQ@CM nanoparticles have been demonstrated to show strong multienzyme activities, which is useful to scavenge the reactive oxygen species and promote the polarization of microglia into the anti-inflammatory M2-like phenotype to relieve neuroinflammation. After the treatment of nanoparticles, all the dopamine level in cerebrospinal fluid, the tyrosine hydroxylase, and the ionized calcium binding adapter protein 1 of PD mice recover to normal levels.

### CNS tumors

CNS tumors are one of the most common solid tumors, which consist of intracranial metastases from systemic cancers, meningiomas, lioblastoma, etc. According to the Lancet Neurology, there were 330, 000 incident cases of CNS cancer and 227, 000 deaths globally in 2016, seriously threatening to human life safety [87]. Glioblastoma are the most common malignant tumors of the central nervous system. The patients with brain tumors are at higher risks of neurocognitive decline, depression, fatigue, endocrinopathies, and venous thromboembolism [88]. CNS tumors are most common diseases among children, adolescents, and young adults, with a high prevalence [89]. Nowadays, the surgical resection is still the preferred treatment strategy of patients. However, for those patients with special tumor location, the palliative care usually leads to poor prognosis. In this context, tumor-targeted drugs are continuously being developed for the timely diagnosis and coordination of gliomas [29, 90–92].

**Fig. 4:**
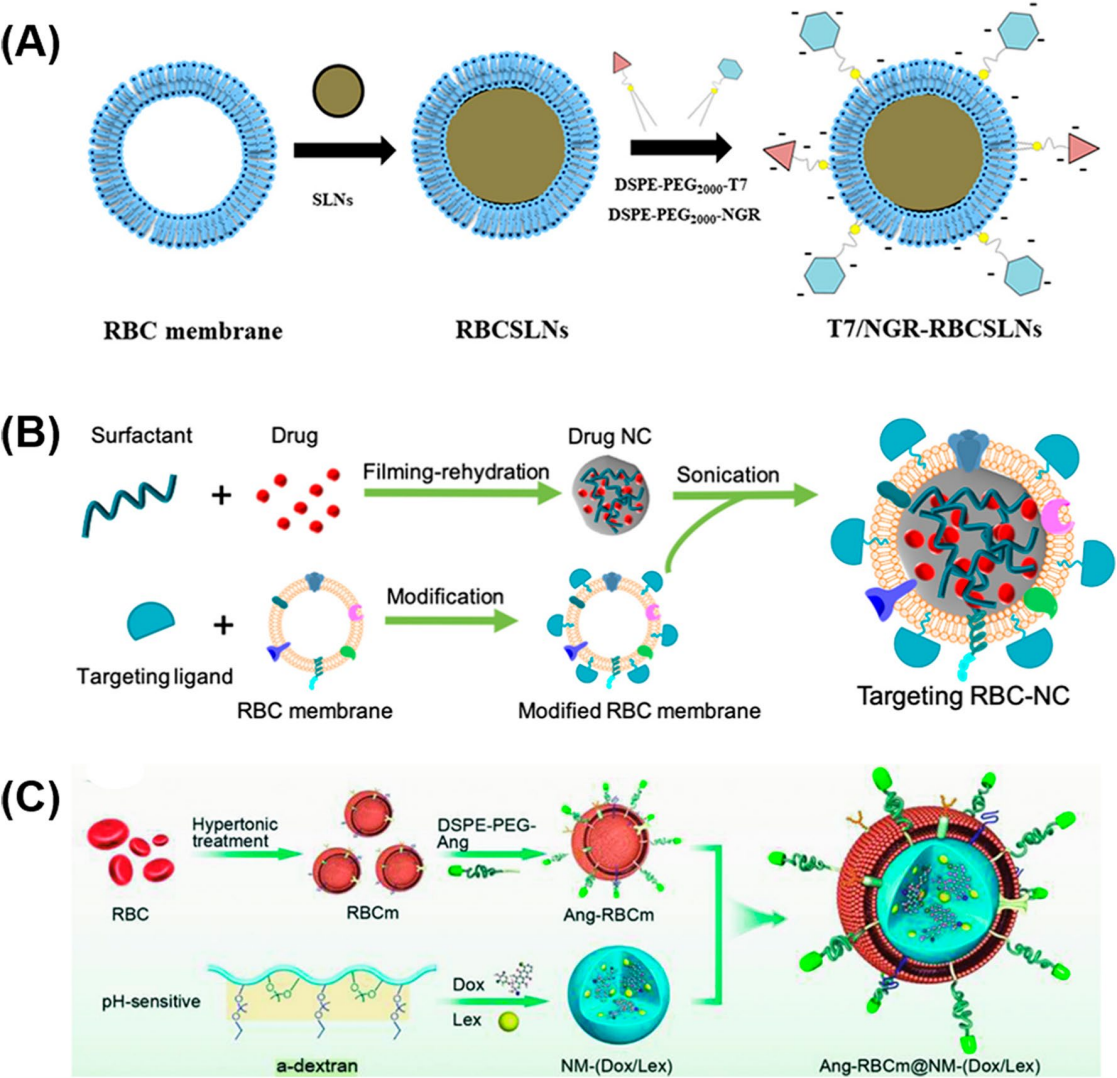
Preparation procedures of RBC membrane-based nanomaterials of T7/NGR-RBCSLNs (**A**), RBC-NC (**B**) and Ang-RBCm@NM-(DOX/Lex) (**C**). Reproduced with permission from Fu et al. [93], Chai et al. [94], Zou et al. [95]

RBC membrane has been used as a common biomimetic material for brain tumor diagnosis and treatment. For example, Chai et al. [34] reported a kind of RBC membrane-coated nanoparticle (RBCNP). The outer RBC membrane of nanoparticles was further modified with neurotoxin-derived peptide ^D^CDX (^D^CDX -RBCNPs) to endow the nanoparticles with highly binding affinity with nicotinic acetylcholine receptors (nAChRs) expressed on the surface of brain endothelial cells. Through a series of in vitro and in vivo studies, it has been verified that ^D^CDX-RBCNPs can penetrate the BBB and show excellent brain target ability. Additionally, the conventional chemotherapeutic drugs, DOX, can be loaded in the ^D^CDX-RBCNPs nanoparticles, which can be delivered into the tumor tissues specifically. In another work, Fu et al. [93] coated solid lipid nanoparticles into red blood cell membrane to obtain the RBCSLN. In addition, T7 peptide (CHAIYPRH) and NGR peptide (CYGGRGNG) were further modified on the RBC membrane (T7/NGR-RBCSLNs) (Fig. 4A). The obtained nano-system has the ability to target glioma through the outer functional cell membrane. It is proved that the prepared dual-modified RBCSLNs encapsulating with vincristine (VCR) could efficiently increase the anticancer effects of VCR both in vitro and in vivo. In addition, Chai et al. [94] proposed an anticancer drug delivery strategy by coating the drug nanocrystals (NCs) with RBC membrane. To efficiently target the tumor, a tumor-targeting ligand c(RGDyK) are introduced on the surface of RBC-NCs (Fig. 4B). The insoluble chemotherapeutic drug docetaxel (DTX) is selected as a model payload to synthesize RBC-NC(DTX). Comparing with NC(DTX), RBC-NC(DTX) can cross the BBB after intravenous administration with higher biosafety. Zou et al. [95] modified the RBC membranes with angiopep-2 (Ang), and coated the pH-sensitive nanomedicine DOX/lexiscan (Lex) with this functionalized RBC membranes, known as Ang-RBCm@NM-(DOX/Lex) (Fig. 4C). The functionalized RBC membranes can endow the nanoparticles with stealth nature in the blood stream, and the nanoparticles have the enough time to penetrate BBB with the help of angiopep-2. After arriving the tumor tissues, the chemotherapeutic drugs DOX and Lex can be successively released at a lower pH, achieving the tumor prohibition.

**Fig. 5:**
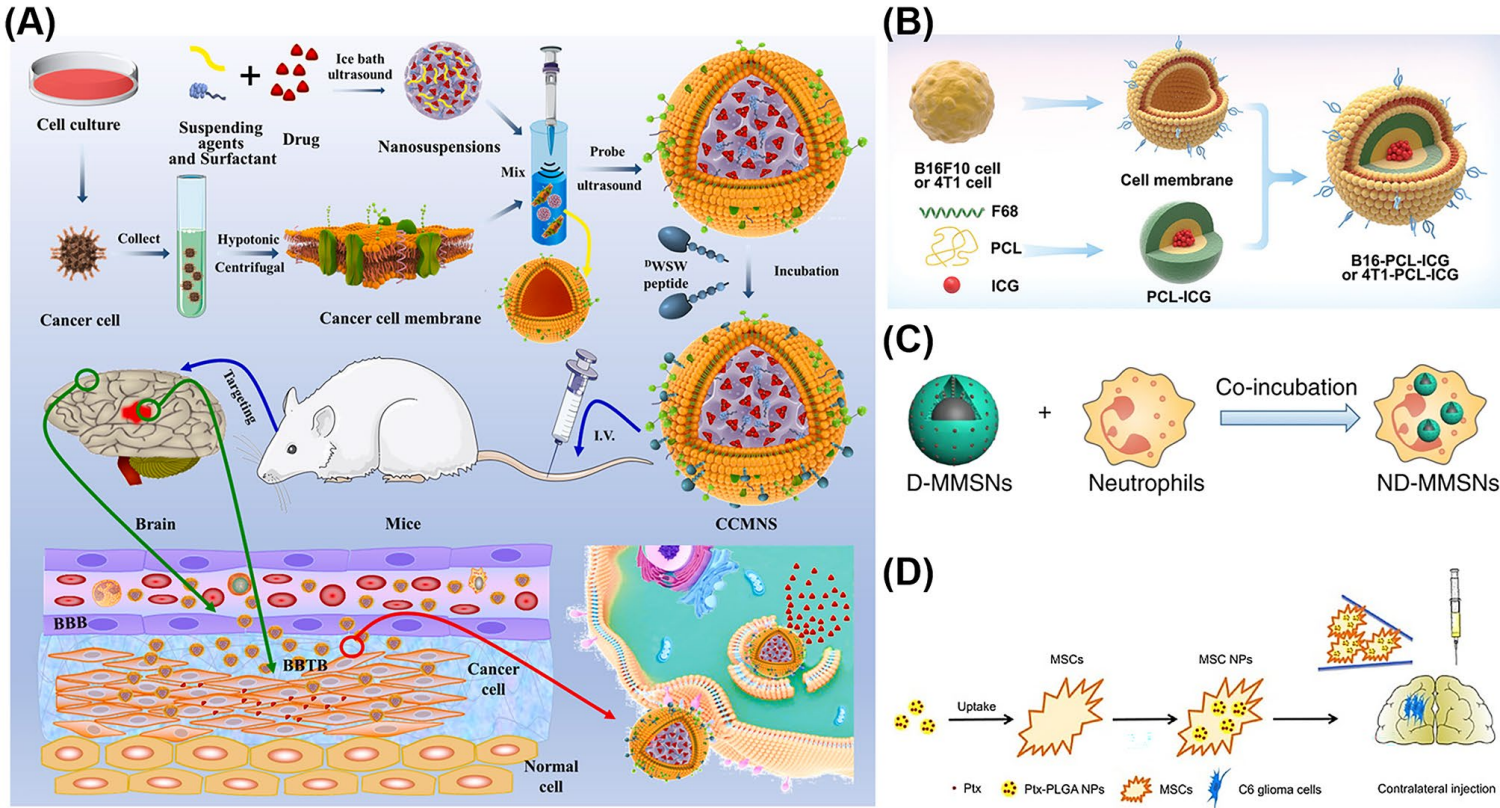
Preparation procedures of cancer cell membrane-based nanomaterials of ^D^WSW-CCM-(PTX)NS (**A**), B16-PCL-ICG or 4T1-PCL-ICG (**B**), ND-MMSNs (**C**) and MSCs-NPs (**D**). Reproduced with permission from Fan et al. [90], Wang et al. [92], Wu et al. [44], Wang et al. [60]

Apart from the membrane of RBCs, cancer cell membrane is also a promising choice for constructing the cell membrane-based nanoparticles for brain tumor theranostics. Han et al. [29] utilized C6 rat glioblastoma cells membrane to encapsulate the polyethylenimine (PEI25k)/plasmid DNA (pDNA) complexes (PEI25k/pDNA/CM) for gene delivery. With the membranes coating, PEI25k/pDNA has improved colloidal stability with negative surface charge. In addition, comparing with no membranes coating (PEI25k/pDNA), PEI25k/pDNA/CM has lower toxicity in vitro and in vivo. Moreover, PEI25k/pDNA/CM has higher cellular uptake and transfection efficiency than the PEI25k/pDNA complexes, thus possessing an enhanced gene delivery efficiency and tumor limitation effects in the intracranial glioblastoma model. Apparently, glioblastoma cells membranes are beneficial to gene delivery into the brain with improved efficiency and reduced toxicity. In another study, Fan et al. [90] utilized homologous targeting mechanism of glioblastoma cells to design a biomimetic drug-delivery systems (^D^WSW-CCM-(PTX)NS), as shown in Fig. 5A. The paclitaxel nanosuspensions ((PTX)NS) is coated with glioma C6 cancer cell membrane (CCM) and the CCM is further modified with the ligand D-type WSW(^D^S^D^Y^D^P^D^G^D^W^D^S^D^W) peptide ^D^WSW. It is proven that as an effective camouflaged nanosuspensions, ^D^WSW-CCM-(PTX)NS can effectively avoid to be cleared by the immune system, and can cross the BBB to selectively target tumor site. In the in vivo glioma treatment experiment, the ^D^WSW-CCM-(PTX)NS could effectively limit the proliferation of glioma cells and obviously prolong the survival period of glioma-bearing mice. In addition, Wang et al. [92] designed a nano-system through encapsulating the polymeric nanoparticles into the brain metastatic tumor cell membrane (B16-PCL-ICG or 4T1-PCL-ICG) (Fig. 5B). In detail, the core of nano-system is indocyanine green (ICG)-loaded polymeric nanoparticles, which are fabricated from poly(caprolactone) (PCL) and Pluronic copolymer F68. The shell membrane is extracted from B16F10 mouse melanoma cells and 4T1 mammary breast cells, respectively. Overall, the nanoparticles protected by the brain metastatic tumor cell membranes show extremely higher ability to cross the intact BBB, prolong blood circulation and enhance tumor accumulation. In addition, with the help of nanoparticles, tumor growth could be significantly inhibited through the photothermal therapy. Tapeinos et al. [96] synthesized a hybrid nanocubes composed of magnetite (Fe_3_O_4_) and manganese dioxide (MnO_2_). The hybrid nanocubes are coated with human glioblastoma cell lines U-251 MG cell-derived membranes to obtain nano-drug named CM-NCubes. The in vitro BBB penetration experiment demonstrated that 75.0% ± 3.7% of the incubated CM-NCubes in the abluminal part of a transwell insert were able to pass through after 48 h, and subsequently be internalized by U-251 MG cells. The CM-NCubes appear to be a promising option as a realistic theranostic platform for glioblastoma after a further investigation in vivo study.

Immune cell membranes, such as macrophage membranes, have also been used to modify nanomaterials for glioma. Xiao et al. [91] developed an immune membrane biomimetic nanoplatform with macrophage membranes. The poly(N-vinylcaprolactam) (PVCL) NGs, loaded with MnO_2_ and cisplatin, are co-encapsulated into macrophage membranes (MPM@P NGs). This nanoplatform can be applied for the MRI diagnosis, and combinational chemotherapy/chemodynamic therapy (CDT) of orthotopic glioma owing to the outstanding tumor targeting ability. The existence of macrophage membrane prolongs the blood circulation time of nanoparticles to ensure the BBB penetration and glioma targeting. Moreover, owing to the presence of endogenous glutathione (GSH) in the tumor microenvironment, the disulfide bond (S-S) inside the nanoparticles could be collapsed for specifically releasing anti-tumor drugs.

In addition to the aforementioned cell membrane-based nanoparticles, directly delivering the nanoparticles through the alive cells is another strategy [42–44, 60]. Guo et al. [42] co-incubated citric-acid coated iron oxide nanoparticles (CIONPs) with BV2 microglial cells, and the resultant nanoparticles-contained cells have been used for intraoperative optical imaging. The BV2 are also loaded with near-infrared fluorescent dye DiD for fluorescence-guided brain tumor surgery. It is confirmed that the nanoparticle-contained cells, known as DiDBV2-Fe, show a strong tumor chemotaxis to monocyte chemoattractant protein-1 (CCL2) excreted by U87MG tumor cells. After administered *via* the carotid artery injection in an orthotopic glioblastoma mouse model, DiDBV2-Fe successfully penetrates the BBB to visualize the activated microglial cells of the brain. Meanwhile, the DiDBV2-Fe can retain in the brain tumor with a prolonged period (4–24 h), which is benefit to time-consuming brain resection operations. In another work, Xue et al. [43] encapsulated paclitaxel (PTX)-loaded liposomes (PTX-CL) into neutrophils to suppress postoperative glioblastoma recurrence (PTX-CL/NEs). PTX-CL/NEs possess the ability to cross the BBB and orientate the inflammation site in the brain. Because of the concentrated activating signals, NETs can be formed and the PTX-CL can be simultaneously released to kill the remaining infiltration tumor cells. This delivery system efficiently decreases the tumor volume and limit the recurrent growth with increasing survival rates.

Wu et al. [44] utilized neutrophils internalization to coat DOX-loaded magnetic mesoporous silica nanoparticles (MMSNs) (ND-MMSNs), as shown in Fig. 5C. After the intravenous injection of ND-MMSNs into the glioma-bearing mice, the nanoparticles-loaded neutrophils will migrate outside the vasculature and accumulate in the inflamed sites of the tumor, followed by the release of D-MMSNs cargos, resulting in precise diagnosis and high anti-glioma efficacy. Wang et al. [60] co-incubated PTX-encapsulated poly(D,L-lactide-co-glycolide) (PLGA) nanoparticles with MSCs. After the nanoparticles were ingested, the MSCs were collected for orthotopic glioma therapy, as shown in Fig. 5D. Because of the characteristic of MSCs that involve inherent tropism towards tumor cells, MSCs-NPs can efficiently target glioma cells and release PTX. In addition, nanoparticle-loaded fluorescent MSCs can be tracked throughout the tumor mass for 2 days in vivo after contralateral injection, and the survival time of tumor-bearing rats can be significantly prolonged in comparison with the controls.

## Conclusion and Outlook

In summary, cell membrane-based nanomaterials can be served as one of the potential candidates for the theranostics of CNS diseases. The cell membrane can endow the conventional nanomaterials with unique biofunction, thereby realizing the precise delivery, strong targeting, multi-modalities diagnosis, and effective treatment of CNS diseases. In addition, the cell membrane can also enhance the biosafety of the conventional nanomaterials. Up to now, many different types of nanomedicines have been increasingly developed with the functionalized membranes, which can be served as theranostic agents for the suitable CNS diseases, and employed in combinatorial approaches for disease treatments. However, the long-term stability of cell membrane-based nanomaterials is still an ongoing and worrisome problem and the validation on large animals such as the non-human primates with cell membrane-based nanomaterials has not further evaluated. Furthermore, there is no effective ways and technologies to synthesize in large quantities of cell membrane-based nanomaterials. Nevertheless, as the emerging biomimetic nanotechnology begins to mature, the clinical translation of these cell membrane-based nanomaterials in the diagnosis and treatment of CNS diseases is still expected.

## Data Availability

The data used to support this review are included within the article.

## References

[1] Gammon K (2014). Neurodegenerative disease: Brain windfall. Nature.

[2] Waris A, Ali A, Khan AU et al. Applications of various types of nanomaterials for the treatment of neurological Disorders. Nanomaterials (Basel). 2022;12:2140. 10.3390/nano12132140 PMC9268720 35807977

[3] Schliebs R, Arendt T (2011). The cholinergic system in aging and neuronal degeneration. Behav Brain Res.

[4] Nguyen TT, Dung Nguyen TT, Vo TK (2021). Nanotechnology-based drug delivery for central nervous system disorders. Biomed Pharmacother.

[5] Gitler AD, Dhillon P, Shorter J (2017). Neurodegenerative disease: models, mechanisms, and a new hope. Dis Models Mech.

[6] Zhang X, Zhou J, Gu Z (2021). Advances in nanomedicines for diagnosis of central nervous system disorders. Biomaterials.

[7] Xie J, Shen Z, Anraku Y (2019). Nanomaterial-based blood-brain-barrier (BBB) crossing strategies. Biomaterials.

[8] Kopec BM, Kiptoo P, Zhao L (2020). Noninvasive brain delivery and efficacy of BDNF to Stimulate Neuroregeneration and suppression of Disease Relapse in EAE mice. Mol Pharm.

[9] Zhao Z, Nelson AR, Betsholtz C (2015). Establishment and dysfunction of the blood-brain barrier. Cell.

[10] Abbott NJ, Patabendige AAK, Dolman DEM (2010). Structure and function of the blood–brain barrier. Neurobiol Dis.

[11] Naqvi S, Panghal A, Flora SJS (2020). Nanotechnology: a Promising Approach for Delivery of neuroprotective drugs. Front Neurosci.

[12] Pajouhesh H, Lenz GR (2005). Medicinal Chemical Properties of successful central nervous system drugs. NeuroRX.

[13] Fischer H, Gottschlich R, Seelig A (1998). Blood-brain barrier permeation: molecular parameters governing Passive Diffusion. J Membr Biol.

[14] Jankovic J. Aguilar LG. Current approaches to the treatment of parkinson’s disease. Neuropsych Dis Treat. 2008;4:743–57. 10.2147/ndt.s2006 PMC2536542 19043519

[15] Nguyen TT, Nguyen TTD, Nguyen TKO (2021). Advances in developing therapeutic strategies for Alzheimer’s disease. Biomed Pharmacother.

[16] Treatment of Alzheimer’s disease. How Is Alzheimer’s disease treated? National Institute on Aging. 2023. https://www.nia.nih.gov/health/how-alzheimers-disease-treated

[17] Poovaiah N, Davoudi Z, Peng H (2018). Treatment of neurodegenerative disorders through the blood–brain barrier using nanocarriers. Nanoscale.

[18] Chen L, Hong W, Ren W (2021). Recent progress in targeted delivery vectors based on biomimetic nanoparticles. Signal Transduct Target Ther.

[19] Zhang P, Li H. Nanoprobes for visualization of Cancer Pathology in vivo. Acta Chim Sinica. 2022;80:805–16.

[20] Zhang P, Meng J, Li Y (2021). Nanotechnology-enhanced immunotherapy for metastatic cancer. The Innovation.

[21] Vilella A, Tosi G, Grabrucker AM (2014). Insight on the fate of CNS-targeted nanoparticles. Part I: Rab5-dependent cell-specific uptake and distribution. J Controlled Release.

[22] Sevencan C, McCoy RSA, Ravisankar P (2020). Cell membrane nanotherapeutics: from synthesis to Applications Emerging Tools for Personalized Cancer Therapy. Adv Ther.

[23] Zhang P, Li Y, Tang W (2022). Theranostic nanoparticles with disease-specific administration strategies. Nano Today.

[24] Wang H, Liu Y, He R (2020). Cell membrane biomimetic nanoparticles for inflammation and cancer targeting in drug delivery. Biomater Sci.

[25] He F, Zhu L, Zhou X (2022). Red blood cell membrane-coated Ultrasmall NaGdF4 nanoprobes for high-resolution 3D magnetic resonance angiography. ACS Appl Mater Interfaces.

[26] Li M, Fang H, Liu Q (2020). Red blood cell membrane-coated upconversion nanoparticles for pretargeted multimodality imaging of triple-negative breast cancer. Biomaterials Sci.

[27] Feng L, Dou C, Xia Y (2021). Neutrophil-like cell-membrane-coated Nanozyme Therapy for ischemic brain damage and long-term neurological functional recovery. ACS Nano.

[28] Zhu C, Ma J, Ji Z et al. Recent advances of cell membrane coated nanoparticles in treating cardiovascular disorders. Molecules. 2021;26:3428. 10.3390/molecules26113428 PMC8201295 34198794

[29] Han S, Lee Y, Lee M (2021). Biomimetic cell membrane-coated DNA nanoparticles for gene delivery to glioblastoma. J Controlled Release.

[30] Liu W, Yan Q, Xia C (2021). Recent advances in cell membrane coated metal–organic frameworks (MOFs) for tumor therapy. J Mater Chem B.

[31] Xia Q, Zhang Y, Li Z (2019). Red blood cell membrane-camouflaged nanoparticles: a novel drug delivery system for antitumor application. Acta Pharm Sinica B.

[32] Suski JM, Lebiedzinska M, Wojtala A (2014). Isolation of plasma membrane–associated membranes from rat liver. Nat Protoc.

[33] Kroll AV, Fang RH, Jiang Y (2017). Nanoparticulate Delivery of Cancer Cell membrane elicits Multiantigenic Antitumor Immunity. Adv Mater.

[34] Chai Z, Hu X, Wei X (2017). A facile approach to functionalizing cell membrane-coated nanoparticles with neurotoxin-derived peptide for brain-targeted drug delivery. J Controlled Release.

[35] Gao C, Chu X, Gong W (2020). Neuron tau-targeting biomimetic nanoparticles for curcumin delivery to delay progression of Alzheimer’s disease. J Nanobiotechnol.

[36] Zhu J, Zhang M, Zheng D (2018). A Universal Approach to render nanomedicine with Biological Identity derived from cell membranes. Biomacromolecules.

[37] Rao L, Cai B, Bu L-L (2017). Microfluidic electroporation-facilitated synthesis of Erythrocyte membrane-coated magnetic nanoparticles for enhanced imaging-guided Cancer Therapy. ACS Nano.

[38] Patty PJ, Frisken BJ (2003). The pressure-dependence of the size of Extruded vesicles. Biophys J.

[39] Israelachvili JN, Mitchell DJ, Ninham BW (1976). Theory of self-assembly of hydrocarbon amphiphiles into micelles and bilayers. J Chem Soc Faraday Trans 2.

[40] Shi M, Shen K, Yang B (2021). An electroporation strategy to synthesize the membrane-coated nanoparticles for enhanced anti-inflammation therapy in bone infection. Theranostics.

[41] Imran M, Jha LA, Hasan N (2022). Nanodecoys” - future of drug delivery by encapsulating nanoparticles in natural cell membranes. Int J Pharm.

[42] Guo L, Zhang X, Wei R (2020). Engineering microglia as intraoperative optical imaging agent vehicles potentially for fluorescence-guided surgery in gliomas. Biomaterials Sci.

[43] Xue J, Zhao Z, Zhang L (2017). Neutrophil-mediated anticancer drug delivery for suppression of postoperative malignant glioma recurrence. Nat Nanotechnol.

[44] Wu M, Zhang H, Tie C (2018). MR imaging tracking of inflammation-activatable engineered neutrophils for targeted therapy of surgically treated glioma. Nat Commun.

[45] Zhou X, Luo B, Kang K (2019). Leukocyte-repelling Biomimetic Immunomagnetic Nanoplatform for High-Performance circulating Tumor cells isolation. Small.

[46] Zou S, Wang B, Wang C (2020). Cell membrane-coated nanoparticles: research advances. Nanomedicine.

[47] Fang RH, Hu C-MJ, Zhang L (2012). Nanoparticles disguised as red blood cells to evade the immune system. Expert Opin Biol Ther.

[48] van der Meijden PEJ, Heemskerk JWM (2019). Platelet biology and functions: new concepts and clinical perspectives. Nat Reviews Cardiol.

[49] Li M, Li J, Chen J (2020). Platelet membrane Biomimetic magnetic nanocarriers for targeted delivery and in situ generation of nitric oxide in early ischemic stroke. ACS Nano.

[50] Xu J, Wang X, Yin H (2019). Sequentially site-specific delivery of thrombolytics and neuroprotectant for enhanced treatment of ischemic stroke. ACS Nano.

[51] Cline MJ, Lehrer RI, Territo MC (1978). Monocytes and macrophages: functions and Diseases. Ann Intern Med.

[52] Joffe AM, Bakalar MH, Fletcher DA (2020). Macrophage phagocytosis assay with reconstituted target particles. Nat Protoc.

[53] Yu Q, Jiang X, Liu X (2022). Glutathione-modified macrophage-derived cell membranes encapsulated metformin nanogels for the treatment of spinal cord injury. Biomaterials Adv.

[54] Tang W, Yang Y, Yang L (2021). Macrophage membrane-mediated targeted drug delivery for treatment of spinal cord injury regardless of the macrophage polarization states. Asian J Pharm Sci.

[55] Murray PJ (2017). Macrophage polarization. Annu Rev Physiol.

[56] Villanueva MT (2017). Chemotherapy: neutrophils deliver the goods. Nat Rev Cancer.

[57] Bi Y, Duan W, Chen J (2021). Neutrophil decoys with anti-inflammatory and anti-oxidative Properties reduce secondary spinal cord Injury and improve neurological functional recovery. Adv Funct Mater.

[58] Zhang X, Yao S, Liu C (2015). Tumor tropic delivery of doxorubicin-polymer conjugates using mesenchymal stem cells for glioma therapy. Biomaterials.

[59] Mendanha D, Vieira de Castro J, Ferreira H (2021). Biomimetic and cell-based nanocarriers – new strategies for brain tumor targeting. J Controlled Release.

[60] Wang X, Gao J, Ouyang X (2018). Mesenchymal stem cells loaded with paclitaxel-poly (lactic-co-glycolic acid) nanoparticles for glioma-targeting therapy. Int J Nanomed.

[61] Roger M, Clavreul A, Venier-Julienne M-C (2010). Mesenchymal stem cells as cellular vehicles for delivery of nanoparticles to brain tumors. Biomaterials.

[62] Fang RH, Kroll AV, Gao W (2018). Cell Membrane Coating Nanotechnology Advanced Materials.

[63] Fang H, Li M, Liu Q (2020). Ultra-sensitive nanoprobe modified with Tumor Cell membrane for UCL/MRI/PET Multimodality Precise Imaging of Triple-Negative breast Cancer. Nano-Micro Lett.

[64] Zenaro E, Pietronigro E, Bianca VD (2015). Neutrophils promote Alzheimer’s disease–like pathology and cognitive decline via LFA-1 integrin. Nat Med.

[65] Kumar A, Singh A, Ekavali (2015). A review on Alzheimer’s disease pathophysiology and its management: an update. Pharmacol Rep.

[66] Gauthier S, Webster C, Servaes S, et al. World Alzheimer Report 2022: Life after diagnosis: Navigating treatment, care and support. Alzheimer’s Disease International. 2022. https://www.alzint.org/resource/world-alzheimer-report-2022/

[67] Bagyinszky E, Giau VV, Shim K (2017). Role of inflammatory molecules in the Alzheimer’s disease progression and diagnosis. J Neurol Sci.

[68] Serrano-Pozo A, Frosch MP, Masliah E (2011). Neuropathological alterations in Alzheimer disease. Cold Spring Harbor perspectives in medicine.

[69] Ouyang Q, Meng Y, Zhou W (2022). New advances in brain-targeting nano-drug delivery systems for Alzheimer’s disease. J Drug Target.

[70] Han Y, Chu X, Cui L (2020). Neuronal mitochondria-targeted therapy for Alzheimer’s disease by systemic delivery of resveratrol using dual-modified novel biomimetic nanosystems. Drug Delivery.

[71] Zhao N, Yang X, Calvelli HR (2020). Antioxidant nanoparticles for concerted inhibition of α-Synuclein fibrillization, and attenuation of Microglial Intracellular aggregation and activation. Front Bioeng Biotechnol.

[72] Gao C, Wang Y, Sun J (2020). Neuronal mitochondria-targeted delivery of curcumin by biomimetic engineered nanosystems in Alzheimer’s disease mice. Acta Biomater.

[73] Han Y, Gao C, Wang H (2021). Macrophage membrane-coated nanocarriers co-modified by RVG29 and TPP improve brain neuronal mitochondria-targeting and therapeutic efficacy in Alzheimer’s disease mice. Bioactive Mater.

[74] Jiahui F, Xiaoguang L, Xueying Y (2023). Global burden, risk factors analysis, and Prediction Study of ischemic stroke, 1990–2030. Neurology.

[75] Hankey GJ (2017). Stroke.

[76] Silva GS, Koroshetz WJ, González RG et al. Causes of ischemic stroke. Acute ischemic stroke: Imaging and Intervention. 2011:25–42.

[77] Wang Y, Wang Y, Li S (2021). Functionalized nanoparticles with monocyte membranes and rapamycin achieve synergistic chemoimmunotherapy for reperfusion-induced injury in ischemic stroke. J Nanobiotechnol.

[78] Kumar R, Lim J, Mekary RA (2018). Traumatic spinal Injury: Global Epidemiology and Worldwide volume. World Neurosurg.

[79] Li J, Cai S, Zeng C (2022). Urinary exosomal vitronectin predicts vesicoureteral reflux in patients with neurogenic bladders and spinal cord injuries. Experimental and Therapeutic Medicine.

[80] Cheriyan T, Ryan DJ, Weinreb JH (2014). Spinal cord injury models: a review. Spinal Cord.

[81] Balestrino R, Schapira AHV (2020). Parkinson disease. Eur J Neurol.

[82] Chen F, Chen S, Si A (2022). The long-term trend of Parkinson’s disease incidence and mortality in China and a bayesian projection from 2020 to 2030. Front Aging Neurosci.

[83] Zhang H, Wang Z, Qi S (2020). Awareness, treatment, and Rehabilitation of Elderly with Parkinson’s Disease - China, 2015–2017. China CDC Wkly.

[84] Dorsey ER, Constantinescu R, Thompson JP (2007). Projected number of people with Parkinson disease in the most populous nations, 2005 through 2030. Neurology.

[85] Moni MM, Begum M, Uddin M (2021). Deciphering the role of nanoparticle-based treatment for Parkinson’s Disease. Curr Drug Metab.

[86] Liu H, Han Y, Wang T (2020). Targeting Microglia for Therapy of Parkinson’s disease by using Biomimetic Ultrasmall Nanoparticles. J Am Chem Soc.

[87] Patel AP, Fisher JL, Nichols E (2019). Global, regional, and national burden of brain and other CNS cancer, 1990–2016: a systematic analysis for the global burden of Disease Study 2016. Lancet Neurol.

[88] McFaline-Figueroa JR, Lee EQ (2018). Brain tumors. Am J Med.

[89] Chen EM, Quijano AR, Seo Y-E (2018). Biodegradable PEG-poly(ω-pentadecalactone-co-p-dioxanone) nanoparticles for enhanced and sustained drug delivery to treat brain tumors. Biomaterials.

[90] Fan Y, Cui Y, Hao W (2021). Carrier-free highly drug-loaded biomimetic nanosuspensions encapsulated by cancer cell membrane based on homology and active targeting for the treatment of glioma. Bioactive Mater.

[91] Xiao T, He M, Xu F (2021). Macrophage membrane-camouflaged responsive polymer nanogels enable magnetic resonance imaging-guided Chemotherapy/Chemodynamic therapy of Orthotopic Glioma. ACS Nano.

[92] Wang C, Wu B, Wu Y (2020). Camouflaging nanoparticles with brain metastatic tumor cell membranes: a New Strategy to Traverse blood–brain barrier for imaging and therapy of brain tumors. Adv Funct Mater.

[93] Fu S, Liang M, Wang Y (2019). Dual-Modified Novel Biomimetic Nanocarriers improve targeting and therapeutic efficacy in Glioma. ACS Appl Mater Interfaces.

[94] Chai Z, Ran D, Lu L (2019). Ligand-modified cell membrane enables the targeted delivery of drug nanocrystals to Glioma. ACS Nano.

[95] Zou Y, Liu Y, Yang Z (2018). Effective and targeted human Orthotopic Glioblastoma Xenograft Therapy via a multifunctional biomimetic nanomedicine. Adv Mater.

[96] Tapeinos C, Tomatis F, Battaglini M (2019). Cell membrane-coated magnetic nanocubes with a homotypic targeting ability increase intracellular temperature due to ROS Scavenging and Act as a versatile Theranostic System for Glioblastoma Multiforme. Adv Healthc Mater.

